# Pelvic collateral pathway during endovascular aortoiliac aneurysm repair with internal iliac artery interruption: a retrospective observational study

**DOI:** 10.1186/s12872-020-01764-y

**Published:** 2020-11-11

**Authors:** Satoshi Nishi, Shogo Hayashi, Takuya Omotehara, Shinichi Kawata, Yoshihiro Suematsu, Masahiro Itoh

**Affiliations:** 1 Department of Anatomy, Tokyo Medical University, 6-1-1, Shinjuku, Shinjuku-ku, Tokyo Japan; 2 Department of Cardiovascular Surgery, Tsukuba Memorial Hospital, 1187-299, Kaname, Tsukuba, Ibaraki Japan; 3 Department of Anatomy, Division of Basic Medical Science, Tokai University School of Medicine, 143, Shimokasuya, Isehara, Kanagawa Japan

**Keywords:** Internal iliac artery, Obturator artery, Medial femoral circumflex artery, Lateral femoral circumflex artery, Collateral circulation, Endovascular aneurysm repair

## Abstract

**Background:**

Ipsilateral branches of the deep femoral artery (DFA) are qualitatively identified as collateral arteries based on angiography after internal iliac artery (IIA) interruption. The purpose of this study was to quantitatively identify the major collateral pathway after unilateral IIA interruption during endovascular aortoiliac aneurysm repair to preserve the pelvic circulation and reduce the risk of ischemic complications.

**Methods:**

The study population included 28 patients (mean age 76.3 years) with aortoiliac aneurysm who underwent endovascular aneurysm repair with unilateral IIA interruption from August 2012 to January 2020. The diameters of the bilateral preoperative and postoperative DFA, lateral femoral circumflex artery (LFCA), medial femoral circumflex artery (MFCA) and obturator artery (ObA) were measured on contrast-enhanced computed tomography using a 3-dimensional image analysis system. The measured values were evaluated and analyzed with a repeated measures two-way analysis of variance and Dunnett’s test.

**Results:**

The postoperative diameters of the MFCA (*P* = 0.051) and ObA (*P* = 0.016) were observed to be larger than the preoperative diameters. Such increases in the MFCA (*P* < 0.001) and ObA (*P* < 0.001) diameters were only found to be significant on the unilateral side of the IIA interruption, and the diameter of the ipsilateral LFCA (*P* < 0.001) was also found to have significantly increased in size. However, no significant arterial extension was found on the contralateral side.

**Conclusions:**

The ipsilateral MFCA-ObA pathway might therefore be a major collateral pathway arising from the DFA to preserve pelvic circulation after unilateral IIA interruption.

## Background

The internal iliac artery (IIA) is the major blood supply to the pelvic organs and buttock muscles. The IIA can also provide collateral circulation to the distal spinal cord and the lumbosacral nerve roots via the iliolumbar and lateral sacral arteries [1]. The IIA is divided into two parts. The superior gluteal artery, which supplies to the upper two thirds of the gluteus maximus muscle and overlying skin, originates from the posterior division of the IIA. The inferior gluteal artery, which supplies the lower part of the gluteus maximus, and the obturator artery (ObA) which originates from the anterior division of the IIA [2].

With an interruption of the unilateral or bilateral IIA, the femoral artery (FA) can also play an important role in buttock and perineal perfusion. The lateral femoral circumflex artery (LFCA) and medial femoral circumflex artery (MFCA) originating from the deep femoral artery (DFA) or FA provide this collateral pathway to the pelvic girdle. The MFCA terminates in the ObA, a branch of the IIA, whereas the LFCA directly supplies the gluteal muscles of the buttocks [3].

Interventional occlusion of the IIA is commonly performed in patients undergoing endovascular aortic aneurysm repair, especially when the aneurysmal process extends to one or both of the iliac artery bifurcations. Although buttock claudication which is a particularly frequent complication of the IIA interruption is often ignored or considered benign by clinicians, buttock claudication may lead to a severe quality of life impairment when it does not regress during follow-up [4].

It has been reported that the major source of collateral supply of an interrupted unilateral IIA comes predominantly from the ipsilateral circumflex branches of the external iliac artery (EIA), FA and DFA, rather than the contralateral IIA [5]. Although this collateral pathway can be qualitatively well-found on computed tomography (CT) angiography or angiography by a vascular surgeon or interventional radiologist [6], only one study has reported the quantitative and statistical identification of the collateral pathway after unilateral IIA interruption [5], and no studies have investigated which of the collateral pathways the medial or lateral circumflex branches of the femoral and deep femoral artery is dominant. The purpose of the present study was to quantitatively identify the major collateral pathway after unilateral IIA interruption during endovascular aortoiliac aneurysm repair in order to preserve the pelvic circulation and reduce the risk of ischemic complications.

## Materials and methods

This retrospective observational study was approved by the Institutional Review Boards of Tokyo Medical University (Approval No. T2019-0097) and Tsukuba Memorial Hospital (Approval No. H31-01-05). Informed consent was obtained from all patients using the opt-out method because of the retrospective design.

**Fig. 1:**
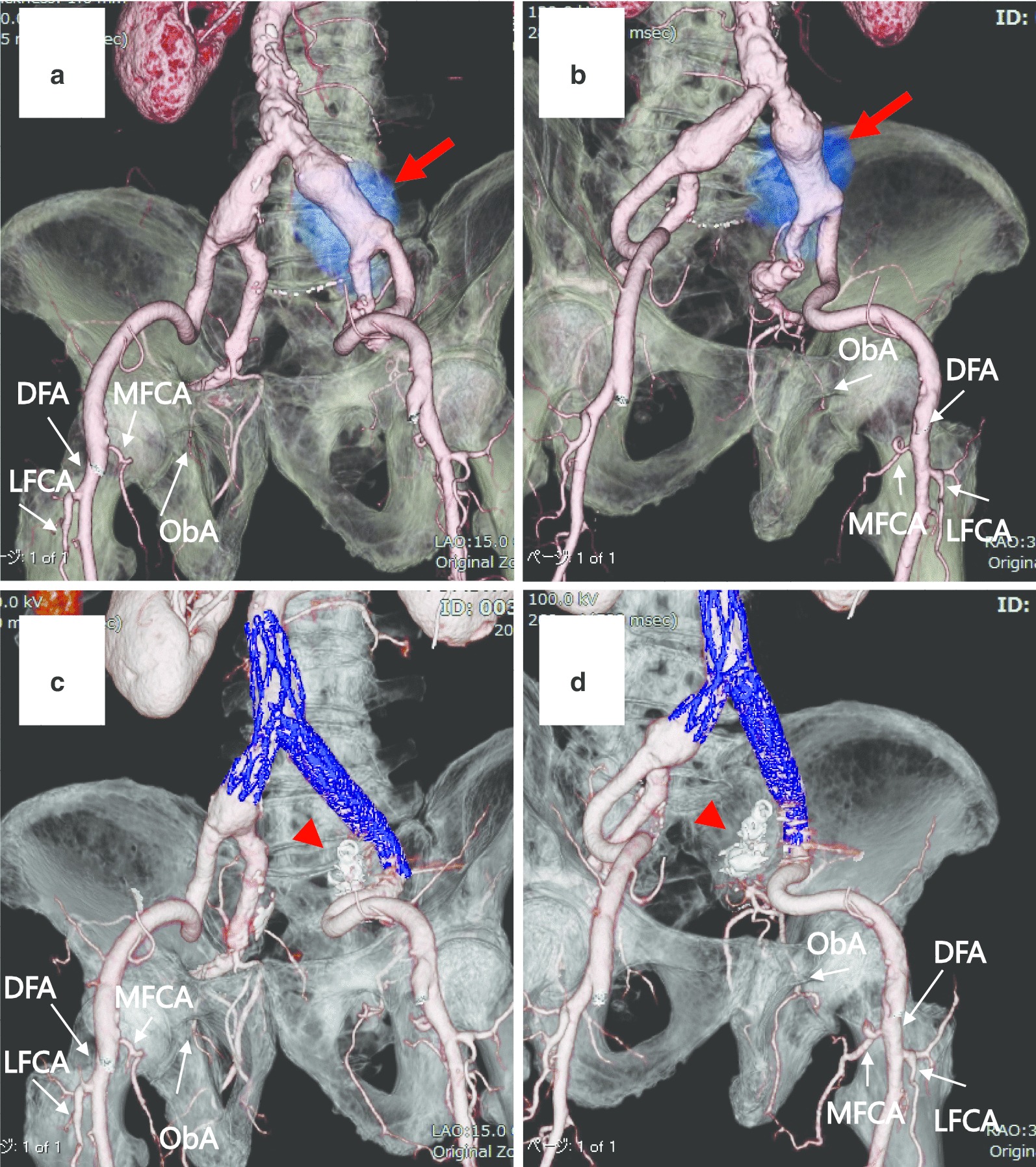
3DCTA in a case of isolated CIA aneurysm. The bilateral superficial femoral arteries were deleted in the all Figures. **a** Preoperative 3DCTA showing an isolated left CIA aneurysm (red arrow), the contralateral DFA, LFCA, MFCA and ObA. **b** Preoperative 3DCTA showing an isolated left CIA aneurysm (red arrow), the ipsilateral DFA, LFCA, MFCA and ObA. **c** Postoperative 3DCTA showing the exclusion of the left CIA aneurysm using a stent graft with left IIA interruption (arrowhead), the contralateral DFA, LFCA, MFCA and ObA. **d** Postoperative 3DCTA showing the exclusion of the left CIA aneurysm using a stent graft with left IIA interruption (arrowhead), the ipsilateral DFA, LFCA, MFCA and ObA, and the ipsilateral MFCA communicated with ipsilateral ObA. *3DCTA* three-dimensional contrast-enhanced computed tomography angiography, *CIA* common iliac artery, *DFA* deep femoral artery, *LFCA* lateral femoral artery, *MFCA* medial femoral circumflex artery, *ObA* obturator artery, *IIA* internal iliac artery

Patients with aortoiliac aneurysms who underwent endovascular aneurysm repair with IIA interruption were investigated. All patients underwent preoperative and postoperative contrast-enhanced CT (Fig. 1). The diameters of the bilateral preoperative and postoperative DFA, LFCA, MFCA and ObA were measured on contrast-enhanced CT using the SYNAPSE VINCENT^Ⓡ^ 3-dimensional image analysis system (FUJIFILM Medical Co., Ltd., Tokyo). The diameter of the DFA, LFCA and MFCA were measured as the maximum diameter within 10 mm from the origin. The diameter of the ObA was measured before the bifurcation of the anterior branch of the ObA. The measured values were evaluated and analyzed with a repeated measures two-way analysis of variance (ANOVA) and Dunnett’s test using the GraphPad Prism software program version 8.4.2 for Macintosh (GraphPad Software, San Diego, California USA, www.graphpad.com). The preoperative contralateral side was used as a control group for Dunnett’s tests. *P* values < 0.05 were considered to be statistically significant. In addition, to investigate whether the values were affected by the position of the origin of the MFCA and LFCA, *t*-tests were performed by grouping the data by the orders of the MFCA and LFCA (the MFCA originating above the LFCA versus the LFCA originating above the MFCA).

## Results

**Fig. 2:**
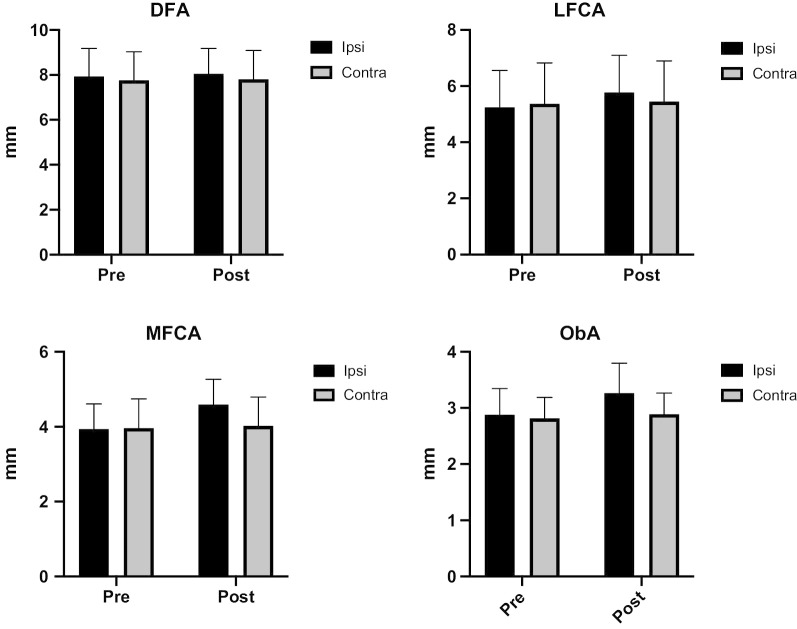
Preoperative and postoperative diameters of the DFA, LFCA, MFCA and ObA on the ipsilateral and contralateral side. Data are expressed as the mean ± standard deviation. *Pre* preoperative, *post* postoperative, *DFA* deep femoral artery, *LFCA* lateral femoral circumflex artery, *MFCA* medial femoral circumflex artery, *ObA* obturator artery, *Ipsi* ipsilateral, *Contra* contralateral

**Fig. 3:**
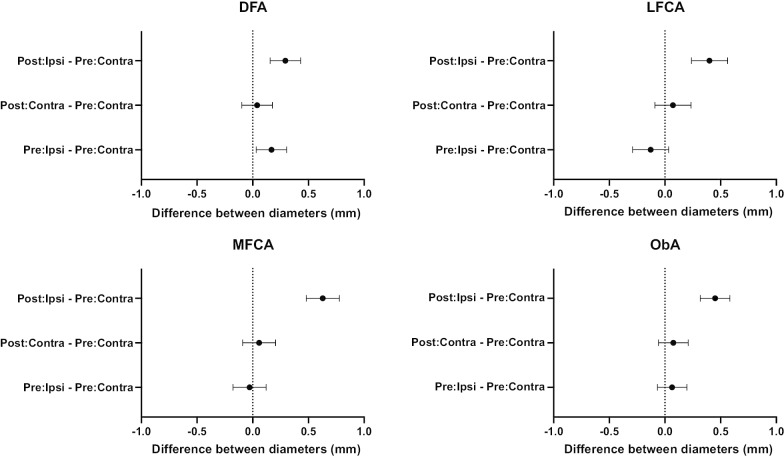
95% confidence intervals determined by Dunnett’s test. Each plot shows the mean (filled circle) and 95% confidence interval (error bar). On the ipsilateral side, the diameters of the LFCA, MFCA and ObA were significantly extended after unilateral internal iliac artery interruption, whereas no arteries were significantly extended on the contralateral side. *DFA* deep femoral artery, *LFCA* lateral femoral circumflex artery, *MFCA* medial femoral circumflex artery, *ObA* obturator artery, *Post* postoperative, *Pre* preoperative, *Ipsi* ipsilateral, *Contra* contralateral

Thirty-six patients with aortoiliac aneurysms underwent endovascular aneurysm repair with unilateral IIA interruption from August 2012 to January 2020. Seven patients (19.4%) who had unilateral (n = 6; 16.7%) or bilateral (n = 1; 2.7%) corona mortis and one patient (2.7%) who did not have a unilateral ObA were excluded. Twenty-eight patients (male, n = 24; female, n = 4; mean age, 76.3 years; range 49–89 years) with an aortoiliac aneurysm (n = 12), an isolated common iliac artery (CIA) aneurysm (n = 10), an isolated IIA aneurysm (n = 3) and an abdominal aortic aneurysm (n = 3), who underwent endovascular aneurysm repair with unilateral IIA interruption were investigated. Ten patients underwent IIA interruption with a plug, 14 patients IIA interruption with coils and 4 patients underwent internal iliac artery coverage which was performed with an implanted stent graft without a plug or coils. The mean interval between the operation and postoperative contrast-enhanced CT was 13.1 days (range 3–161 days). Follow-up contrast-enhanced CT is basically performed within 7 days after aortoiliac endovascular repair at our institution. In patients with renal dysfunction, follow-up contrast-enhanced CT is not performed. In the present study, 26 patients underwent follow-up contrast-enhanced CT not later than postoperative day 30 (range 3–18 days). Two patients with renal dysfunction underwent contrast-enhanced CT for reasons other than follow-up on postoperative day 30 or 161. A repeated measures two-way ANOVA revealed that the diameters of the MFCA (*P* = 0.051) and ObA (*P* = 0.016) exceeded the preoperative values after unilateral IIA interruption, whereas no significant extensions were observed with DFA (*P* = 0.201) and LFCA (*P* = 0.734) (Fig. 2). The results of Dunnett’s tests revealed that on the ipsilateral side, the diameters of the LFCA (*P* < 0.001), MFCA (*P* < 0.001) and ObA (*P* < 0.001) were significantly extended after unilateral IIA interruption, whereas no arteries were significantly extended on the contralateral side (Fig. 3).

**Fig. 4:**
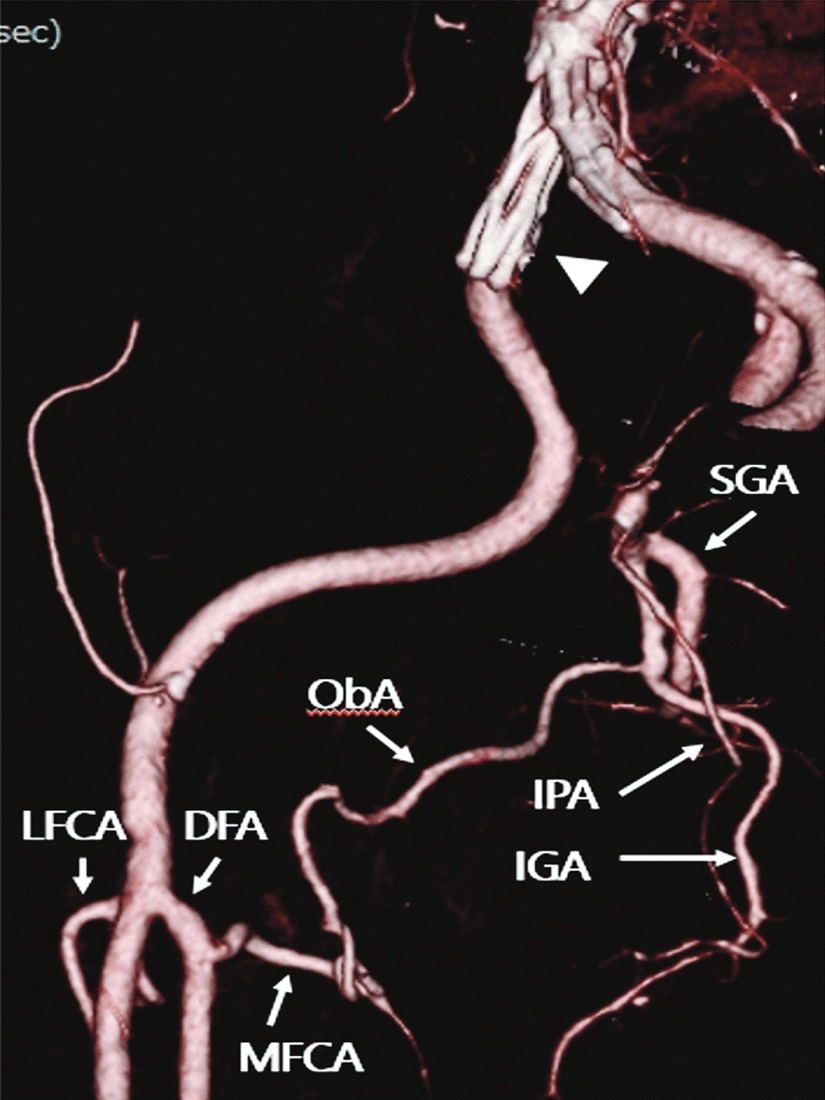
3DCTA after endovascular aneurysm repair with internal iliac artery coverage (arrowhead). 3DCTA shows the ipsilateral MFCA-ObA pathway arising from the DFA which communicated with the IGA. *3DCTA* three-dimensional contrast-enhanced computed tomography angiography, *DFA* deep femoral artery, *LFCA* lateral femoral circumflex artery, *MFCA* medial femoral circumflex artery, *ObA* obturator artery, *SGA* superior gluteal artery, *IGA* inferior gluteal artery, *IPA* internal pudendal artery

The ipsilateral MFCA-ObA pathway arising from the DFA was often found when both the MFCA and ObA were completely preserved during endovascular repair (Fig. 4). In addition, the origin of the MFCA on the ipsilateral side varied; the origin was the DFA in 23 patients (82.1%), the FA in 3 patients (10.7%), and the inferior epigastric artery in 2 patients (7.1%). The origin of the LFCA also varied on the ipsilateral side; the origin was the DFA in 20 patients (71.4%) and the FA in 8 patients (28.6%). The MFCA originating above the LFCA in the ipsilateral side was seen in 14 patients (50.0%). The origin of the MFCA was not significantly associated with the above results (*P* = 0.98).

## Discussion

We showed that the diameter of the MFCA tended to extend and that the diameter of the ObA was significantly extended after unilateral IIA interruption, regardless of the position of the MFCA or LFCA. The MFCA and ObA sometimes play a role as a bypass between the DFA and the gluteal artery when the MFCA and ObA are totally preserved during endovascular repair. Our study suggested that the ipsilateral MFCA-ObA pathway might be a major collateral pathway arising from the DFA to preserve pelvic circulation after unilateral IIA interruption that does not depend on the origins of the MFCA or LFCA. Previous studies reported the incidence of MFCA which originated from the DFA to range from 64.6 to 81% [7]. In the present study, we found that the MFCA originated from the DFA in 82.1% of patients, from the common FA in 10.7% and from the inferior epigastric artery in 7.1%. Regarding the pattern of the LFCA, Üzel et al. reported that the LFCA originated from the DFA (77.3%) and the FA (19.1%) [8]. In our study, LFCA originated from the DFA in 71.4% of patients and the FA in 28.6% of patients. Łabętowicz et al. also reported that the MFCA origin above the LFCA was 63% [9], while the MFCA origin above the LFCA was 50.0% in the present study.

Approximately 20% of the patients presenting with abdominal aortic aneurysms have concomitant iliac artery aneurysms [10]. To treat aortoiliac aneurysms, isolated CIA aneurysms or IIA aneurysms, unilateral or bilateral IIA interruptions are sometimes required. Karch et al. [11] reported that 16% of the patients who underwent endovascular abdominal aortic aneurysm repair intentionally underwent IIA interruption. Buttock claudication, erectile dysfunction and colonic ischemia are more common ischemic complications related to IIA interruption, whereas spinal cord ischemia, gluteal compartment syndrome, bladder dysfunction, decubitus ulcer, and genital ulceration are less common complications [12]. In addition, ligation of the bilateral internal iliac arteries can lead to severe pelvic ischemia with hip and buttock claudication, bladder and bowel dysfunction, colon ischemia, and decubitus ulcer formation [13]. The incidence of buttock claudication was 28% in patients who underwent unilateral IIA interruption and 42% in patients who underwent bilateral IIA interruption during endovascular aneurysm repair [12]. However, Mehta et al. suggested that unilateral and bilateral IIA interruption is a relatively safe procedure, particularly when pelvic collateral circulation from the external iliac and femoral arteries is preserved [10].

There have been some previous studies on the collateral pathways of pelvic circulation. Flanigan et al. and Pierce et al. reported that the circumflex branches of the EIA, FA and DFA provided important collateral perfusion to maintain pelvic circulation [14, 15]. Furthermore, Iliopoulos et al. showed that the blood pressure within an acutely occluded IIA was maintained by collateral perfusion from the ipsilateral EIA and FA and not collateral flow from the contralateral IIA [5]. Additionally, we reported that the MFCA and ObA might be the major collateral pathway that develops after unilateral IIA interruption during endovascular aneurysm repair.

Based on previous studies regarding pelvic collateral circulation, various procedures to preserve pelvic circulation have been reported. Kritpracha et al. reported that IIA coil embolization should be performed as proximally as possible to prevent interference with pelvic collateral circulation [16]. Resnick et al. reported that the Amplatzer vascular plug allowed for consistent preservation of the IIA divisional bifurcation and might allow for preservation of important pelvic collateral flow [17]. Bosanquet et al. [18] showed that while both options were technically possible, plugs could be considered preferential to coils, and could be placed as proximally in the IIA as possible. Chitragari et al. also showed that ligation of the internal iliac arteries was preferred to embolization, and proximal embolization should be preferred to distal embolization to reduce the risk of ischemic complications [19]. On the other hand, IIA bypass during endovascular aneurysm repair preserved the pelvic circulation and reduced the risk of pelvic ischemic complications [20]. Furthermore, either the sandwich technique [21] or iliac branch endoprosthesis [12, 22–24] was performed in total endovascular aortic repair with preservation of the IIA for aortoiliac aneurysms. Recently, a few studies have reported that IIA coverage during endovascular aneurysm repair without embolization was effective for preventing severe pelvic ischemic complications [19, 25].

However, proximal coil embolization or plug embolization of the IIA, IIA bypass, sandwich technique, iliac branch endoprosthesis or IIA coverage during endovascular aortic repair are not necessarily adapted for aortoiliac aneurysms. Tielliu et al. reported that only 52% of patients with aortoiliac or solitary iliac aneurysms were morphologically suitable for iliac branch endoprothesis [26].

The present study suggested that the ObA was one of the most important arteries and that the ipsilateral MFCA-ObA pathway might be an anatomically important collateral pathway to preserve pelvic circulation during unilateral IIA interruption. Abderhalden et al. controlled type II endoleak after endovascular aortoiliac aneurysm repair with ligation of unilateral IIA by coil embolization of the ObA and MFCA via an antegrade ipsilateral FA access [6]. This indicated that blood flowed from the DFA to the ObA through the MFCA after unilateral IIA interruption. Because the ObA, inferior gluteal artery and internal pudendal artery often originate from the anterior division of the IIA, preservation of the orifice of the ObA during IIA interruption can preserve the blood flow of the inferior gluteal artery and the internal pudendal artery, which was supplied from the ipsilateral DFA through the MFCA-ObA pathway and which may reduce the risk of buttock claudication or impotence. One of the reasons why the proximal embolization of the IIA and IIA coverage during endovascular aortic repair are effective techniques for reducing ischemic complications may be that in addition to the LFCA and MFCA, the ObA is often preserved with those techniques. Mehta et al. reported that the iliac and femoral circumflex branches were routinely preserved during the exposure of these vessels [27]. Resnick et al. [17] suggested the importance of preserving the IIA divisional bifurcation to preserve important pelvic collateral flow. Additionally, Yano et al. reported that stenosis of the patent IIA and diseased ascending branches of the ipsilateral DFA were risk factors for pelvic ischemia during IIA interruption [28]. Preservation of the LFCA and MFCA is not technically difficult, however, preservation of the IIA divisional bifurcation is sometimes difficult in cases of endovascular aortoiliac aneurysm repair. When the IIA divisional bifurcation cannot be preserved, preservation of the bifurcation of the ObA originating from the superior or inferior gluteal artery should be considered. Preservation of the MFCA-ObA-gluteal artery pathway is anatomically important for preserving pelvic circulation during IIA interruption.

## Limitations

Several limitations associated with the present study warrant mention. For example, we did not evaluate the changes over time. It is thus possible that new collateral vessels developed or hemodynamic changes occurred later in the postoperative period. While there were only two cases in which contrast-enhanced CT was performed beyond 30 days, further comparisons between the early and late postoperative periods should be made by increasing the number of cases in the future. In addition, cases of corona mortis in which the ObA originated from the inferior epigastric artery were excluded. Further research to compare the incidence and the degree of ischemic complications after unilateral IIA interruption with or without corona mortis is needed. Without the cases of corona mortis, a prospective study to compare ischemic complications after unilateral IIA interruption with and without preservation of the MFCA-ObA-gluteal artery pathway is needed.

## Conclusions

The diameters of the ObA on the ipsilateral and contralateral side significantly expanded after unilateral IIA interruption, the diameters of the MFCA on the ipsilateral and contralateral side tended to expand after unilateral IIA interruption, and the diameters of the ipsilateral LFCA, MFCA and ObA significantly expanded after unilateral IIA interruption. The preoperative assessment of the origin of the LFCA, MFCA and especially ObA is therefore important when IIA interruption is required during endovascular aortoiliac aneurysm repair.

## Data Availability

The datasets used and/or analyzed during the current study are available from the corresponding author on reasonable request.

## Abbreviations

DFA: Deep femoral artery
IIA: Internal iliac artery
LFCA: Lateral femoral circumflex artery
MFCA: Medial femoral circumflex artery
ObA: Obturator artery
FA: Femoral artery
EIA: External iliac artery
CT: Computed tomography
CIA: Common iliac artery
ANOVA: Two-way analysis of variance
